# Computational genomics at BGRS\SB-2016: introductory note

**DOI:** 10.1186/s12864-016-3350-6

**Published:** 2016-12-28

**Authors:** Yuriy L. Orlov, Ancha V. Baranova, Ralf Hofestädt, Nikolay A. Kolchanov

**Affiliations:** 1 Institute of Cytology and Genetics SB RAS, Lavrentyeva, 10, 630090 Novosibirsk, Russia; 2 Novosibirsk State University, Pirogova, 2, 630090 Novosibirsk, Russia; 3 School of Systems Biology, George Mason University, Fairfax, VA 22030 USA; 4 Research Centre for Medical Genetics, Moskvorechie, 1, Moscow, Russia; 5 Bielefeld University, D-33501 Bielefeld, Germany

This special BMC Genomics BGRS\SB-2016 issue continues the series of BioMed Central special post-conference journal issues after BGRS\SB-2016 conference which took place at August 29–September 2, 2016 in Novosibirsk, Russia. This issue, as well as BMC Genetics [1], BMC Plant biology [2], BMC Evolutionary biology and BMC Systems Biology issues collate the papers presented at the Tenth International Conference “Bioinformatics of Genome Regulation and Structure\Systems Biology” (BGRS\SB-2016). The biannual BGRS conference series has long history starting from 1998 in Novosibirsk (http://conf.bionet.nsc.ru/bgrssb2016/archive/). The Institute of Cytology and Genetics of Siberian Branch of the Russian Academy of Sciences (ICG SB RAS) held the conference focusing on systems biology and bioinformatics topics, gene network analysis, post-genomics and sequencing technologies. Since first meeting in 1998 gathering several bioinformatics professionals in Siberia BGRS series has grown to large international event joining medical doctors, mathematicians, biologists in the frames of a multi-conference.

In 2016, the BGRS held several parallel events and symposia: the international Symposium “Systems Biology and Biomedicine” (SBioMed-2016) (http://conf.bionet.nsc.ru/ishg2016/en/), Symposium “Cognitive Sciences, Genomics and Bioinformatics” (CSGB-2016) (http://physiol.ru/csgb2016/), and the Second International Conference on the Mathematical Modeling and High-Performance Computing in Bioinformatics, Biomedicine and Biotechnology (MM-HPC-BBB-2016) (http://conf.bionet.nsc.ru/mm-hpc-bbb-2016/en/). The BGRS Program Committee has collaborated with BioMed Central on full-text thematic issues since 2014. In recent years BioMed Central had published several special issues based on best materials presented at the conference in BMC Genomics (http://www.biomedcentral.com/bmcgenomics/supplements/15/S12), BMC Genetics [3], BMC Evolutionary Biology (http://www.biomedcentral.com/bmcevolbiol/supplements/15/S1), and BMC Systems biology (http://www.biomedcentral.com/bmcsystbiol/supplements/9/S2).

Special issues on bioinformatics were published at the “Journal of Bioinformatics and Computational Biology” [4] and “Vavilov Journal of Selection and Breeding” (http://www.bionet.nsc.ru/vogis/2014-year/18-4-2/) (in Russian).

Current issue of BMC Genomics presents reports on computational genomics discussed at the BGRS\SB-2016 conference.

The paper by A.V. Snezhkina et al. [5] opens this special issue by analysis of differential alternative splicing in colorectal cancer by RNA-Seq data. Using TCGA RNA-Seq datasets derived from colorectal cancer and adjacent normal tissue the authors examined the expression of a thousand of alternative mRNA isoforms involved in cell energy metabolism and found genes with differentially expressed alternative transcripts. Thus a set of tumor-specific mRNA isoforms may be used for cancer diagnosis and treatment methods development.

The work by Kural et al. [6] considers molecular genomics models of aging. In culturing normal diploid cells, senescence may either happen naturally, in the form of replicative senescence, or it may be a consequence of external challenges such as oxidative stress. The authors present a comparative analysis aimed at reconstruction of molecular cascades specific for replicative and stress-induced senescence in human fibroblasts. The promoters of genes differentially expressed in these senescence cell models are unusually enriched by the binding sites for homeobox family proteins, with particular emphasis on HMX1, IRX2, HDX and HOXC13. Kural and colleagues also identified Iroquois Homeobox 2 (IRX2) as a master regulator for the secretion of *SPP1*-encoded osteopontin, a stromal driver for tumor growth.

The paper by Chadaeva et al. [7] highlights problem of identification of single nucleotide polymorphisms (SNPs) located in gene promoter regions and related to genetic diseases. The authors combine two computer-based search methods for SNPs (that alter gene expression): Web-service SNP_TATA_Comparator (DNA sequence analysis) and PubMed-based search for science articles on aggressiveness (disease term) using heuristic keywords. Near the known binding sites for TATA-binding protein in human gene promoters, they found aggressiveness-related candidate SNP markers, including rs1143627 (associated with higher aggressiveness in patients undergoing cytokine immunotherapy), rs544850971 (higher aggressiveness in old women taking lipid-lowering medication), and rs10895068 (childhood aggressiveness-related obesity in adolescence with cardiovascular complications in adulthood). These predicted SNPs may become useful for physicians after validation of these candidate markers by clinical protocols. Thus, this paper shows human polymorphism analysis based on genomics data after cancer and cell culture models.

L. A. Fedoseeva and colleagues [8] consider laboratory animal model to study genomic markers of arterial hypertension. The ISIAH rat strain (Inherited stress-induced arterial hypertension) was developed by selection for high systolic arterial blood pressure (SABP) induced by restraint stress. The authors used RNA-Seq approach to perform the comparative adrenal transcriptome profiling in hypertensive and normotensive rats. Multiple differentially expressed genes related to different biological processes and metabolic pathways were detected. Two transcription factor genes (Nr4a3 and Ppard) might be related to the predominant activation of the sympathetic-adrenal medullary axis in the rats studied. The study strongly highlighted the complex nature of the pathogenesis of stress-sensitive hypertension. These data may be useful for identifying the common molecular determinants in different animal models of arterial hypertension, and as a guide for searching of therapeutic targets for pharmacological intervention.

The paper by Voronina et al. [9] presents genomics studies on human pathogens on example of *Mycobacterium bovis* BCG genomes. BCG (Bacille-Calmette-Guérin) vaccine is used broadly in various regions for the prevention of acute forms of childhood tuberculosis as part of the childhood immunization programs. The control of genome stability is relevant for the worldwide BCG vaccine preventing the acute forms of childhood tuberculosis. BCG sub-strains whole genome comparative analysis and revealing the triggers of sub-strains transition were the purpose of our investigation. Whole genome sequencing of three BCG seed lots confirmed the stability of vaccine sub-strain genome. Comparative analysis of three *Mycobacteruim bovis* and nine *M. bovis* BCG genomes shown that differences between “early” and “late” sub-strains BCG genomes were associated with specific prophage profiles. The authors demonstrate the contribution of prophages in genome mosaic structure formation.

The work by A. A. Moskalev et al. [10] consider drosophila model to study ageing process. Transcriptional changes that contribute to the organism’s longevity and prevent the age-dependent decline of biological functions are not well understood. The authors overexpressed pro-longevity gene encoding glutamate-cysteine ligase catalytic subunit (*Gclc*) and analyzed age-dependent changes in transcriptome that associated with the longevity, stress resistance, locomotor activity, circadian rhythmicity, and fertility. The authors reproduced the life extension effect of neuronal overexpression of the Gclc gene and investigated its influence on the age-depended dynamics of transcriptome and biological functions such as fecundity, spontaneous locomotor activity and circadian rhythmicity, as well as on the resistance to oxidative, proteotoxic and osmotic stresses in flies. *Gclc* overexpression slowed down the age-dependent decline of locomotor activity and circadian rhythmicity, and resistance to stress treatments. The study revealed that *Gclc* overexpression induces transcriptional changes associated with the lifespan extension and uncovered pathways that may be associated with the age-dependent decline of biological functions.

The paper by Romanova et al. [11] presents mitochondrial genome studies on other model organisms – amphipods. The evolutionary history and phylogenetic relationships in Baikalian amphipods still remain poorly understood. The architecture of mitochondrial genomes suitable for robust phylogenetic inferences and may provide additional information on the mechanisms of evolution of amphipods in Lake Baikal. Several complete genomes of Baikalian amphipods were obtained by high-throughput sequencing using the Illumina platform. A phylogenetic inference based on the nucleotide sequences of all mitochondrial protein coding genes revealed the Baikalian species to be a monophyletic group relative to the nearest non-Baikalian species with a completely sequenced mitochondrial genome - *Gammarus duebeni*. The authors show that the mitochondrial genomes of Baikalian amphipods display varying genome organization suggesting an intense rearrangement process during their evolution.

Finally, the work by R. Sultanov et al. [12] discusses bacterial genome studies. *Pseudomonas syringae* is a widespread bacterial species that infects almost all major crops. Different *P. syringae* strains use a wide range of biochemical mechanisms, including phytotoxins and effectors of the type III and type IV secretion systems, which determine the specific nature of the pathogen virulence. Strains 1845 (isolated from dicots) and 2507 (isolated from monocots) were selected for sequencing due to their specialization on different groups of plants. The authors compared virulence factors in these and other available genomes of phylogroup 2 to find genes responsible for the specialization of bacteria.

All the works in this special issue might be united based on sequencing data analysis, but differ in model applications – from human studies to laboratory animals, drosophila and bacteria. Returning back to the tenth anniversary BGRS\SB event, note the perspective meetings that might be the start for new conference series.

The CSGB-2016 Symposium on cognitive sciences has presented special issue at IEEE Xplore Digital Library (http://ieeexplore.ieee.org/xpl/mostRecentIssue.jsp?punumber=7587661). “Journal of Bioinformatics and Computational Biology” (http://www.worldscientific.com/worldscinet/jbcb) continues traditional special issues after BGRS\SB-2016 as well as “Journal of Integrative bioinformatics” (https://www.degruyter.com/view/j/jib) presenting papers by the authors discussed earlier at BGRS conference series. The BGRS/SB-2016 was accompanied by a number of satellite events, including already traditional Young Scientists School “Systems Biology and Bioinformatics” (SBB-2016) (http://conf.bionet.nsc.ru/sbb2016/en/) and Open Russian-German workshop on bioinformatics network “Systems computational biology”.

The BGRS\SB-2016 Proceedings including “Genomics, Transcriptomics and Bioinformatics” section are available at the multi-conference web-site: http://www.bionet.nsc.ru/files/2016/conference/BGRS2016.pdf. In addition, special issues on bioinformatics were published at the “Vavilov Journal of Selection and Breeding” (http://vavilov.elpub.ru/jour/) (in Russian).

Next BGRS conference will take place in 2018. Welcome to Novosibirsk!

## References

[1] Orlov YL, Baranova AV, Markel AL (2016). Computational models in genetics at BGRS\SB-2016: Introductory note. BMC Genetics.

[2] Orlov YL, Baranova AV, Salina EA (2016). Computational plant bioscience at BGRS\SB-2016: Introductory note. BMC Plant Biol.

[3] Baranova AV, Orlov YL (2016). The papers presented at 7th young scientists school “systems biology and bioinformatics” (SBB’15): introductory note. BMC Genet.

[4] Orlov YL, Hofestädt RM, Kolchanov NA (2015). Introductory note for BGRS\SB-2014 special issue. J Bioinform Comput Biol.

[5] Snezhkina AV, Krasnov GS, Zaretsky AR, Zhavoronkov A, Nyushko KM, Moskalev AA, Karpova IY, Afremova AY, Lipatova AV, Kochetkov DV, Fedorova MS, Volchenko NN, Sadritdinova AF, Melnikova NV, Sidorov DV, Popov AY, Kalinin DV, Kaprin AD, Alekseev BY, Dmitriev AA, Kudryavtseva AV (2016). Differential expression of alternatively spliced transcripts related to energy metabolism in colorectal cancer. BMC Genomics.

[6] Kural KC, Tandon N, Skoblov M, Kel-Margoulis OV, Baranova AV (2016). Molecular Models of Aging: Comparative Analysis of Gene Signatures in Replicative Senescence and Stress Induced Premature Senescence. BMC Genomics.

[7] Chadaeva IV, Ponomarenko MP, Rasskazov DA, Sharypova EB, Kashina EV, Matveeva MY, Arshinova TV, Ponomarenko PM, Arkova OV, Bondar NP, Savinkova LK, Kolchanov NA (2016). Candidate SNP markers of aggressiveness-related complications and comorbidities of genetic diseases are predicted by a significant change in the affinity of TATA-binding protein for human gene promoters. BMC Genomics.

[8] Fedoseeva LA, Klimov LO, Ershov NI, Alexandrovich YV, Efimov VM, Markel AL, Redina OE (2016). Molecular determinants of the adrenal gland functioning related to stress-sensitive hypertension in ISIAH rats. BMC Genomics.

[9] Voronina OL, Kunda MS, Aksenova EI, Semenov AN, Ryzhova NN, Lunin VG, Gintsburg AL (2016). Mosaic structure of Mycobacterium bovis BCG genomes as a representation of phage sequences’ mobility. BMC Genomics.

[10] Moskalev AA, Shaposhnikov M, Proshkina E, Belyi A, Fedintsev A, Zhikrivetskaya S, Guvatova Z, Sadritdinova A, Zhavoronkov A, Snezhkina A, Krasnov G, Kudryavtseva AV (2016). The influence of pro-longevity gene Gclc overexpression on the age-dependent changes in Drosophila transcriptome and biological functions. BMC Genomics.

[11] Romanova EV, Aleoshin VV, Kamaltynov RM, Mikhailov KV, Logacheva MD, Sirotinina EA, Gornov AY, Anikin AS, Sherbakov DY (2016). Evolution of mitochondrial genomes in Baikalian amphipods. BMC Genomics.

[12] Sultanov RI, Arapidi GP, Vinogradova SV, Govorun VM, Luster DG, Ignatov AN (2016). Comprehensive analysis of draft genomes of two closely related pseudomonas syringae phylogroup 2b strains infecting mono- and dicotyledon host plants. BMC Genomics.

